# Selenourea: a convenient phasing vehicle for macromolecular X-ray crystal structures

**DOI:** 10.1038/srep37123

**Published:** 2016-11-14

**Authors:** Zhipu Luo

**Affiliations:** 1 Synchrotron Radiation Research Section, National Cancer Institute, Argonne National Laboratory, Argonne, 60439, USA

## Abstract

Majority of novel X-ray crystal structures of proteins are currently solved using the anomalous diffraction signal provided by selenium after incorporation of selenomethionine instead of natural methionine by genetic engineering methods. However, selenium can be inserted into protein crystals in the form of selenourea (SeC(NH_2_)_2_), by adding the crystalline powder of selenourea into mother liquor or cryo-solution with native crystals, in analogy to the classic procedure of heavy-atom derivatization. Selenourea is able to bind to reactive groups at the surface of macromolecules primarily through hydrogen bonds, where the selenium atom may serve as acceptor and amide groups as donors. Selenourea has different chemical properties than heavy-atom reagents and halide ions and provides a convenient way of phasing crystal structures of macromolecules.

Because of the availability of large number of structures of macromolecules stored in the Protein Data Bank^1^ (PDB), the majority of X-ray crystal structures of proteins and nucleic acids are nowadays solved by the Molecular Replacement technique. However, the crystal structures containing molecules for which there is no sufficiently similar atomic model available, have to be solved by the “special atom” method. The initial phasing of diffraction data is then based on the isomorphous signal of heavy atoms or the anomalous signal of certain atoms present in crystals of the native molecules or introduced into investigated crystals^2^.

The “classic” approach, used since the early days of protein crystallography, involves derivatization of native crystals by prolonged soaking in solutions or co-crystallization with various reagents containing heavy metals^3^, such as Hg, Pt, Au etc. Variations of this approach involve, for example, the use of the heavy-metal clusters^4^, especially suitable for structures of very large macromolecular complexes, the gaseous xenon or krypton pressurized into native crystals^5^, or the short soaking in salts of halides^6^ (Br or I). It is also possible to obtain useful anomalous phasing signal from sulfur of Cys and Met naturally occurring in proteins^7^,^8^,^9^ or from phosphorus in nucleic acids^10^.

The currently most widely used approach is based on the introduction of selenomethionine into proteins by genetic engineering methods^11^. Selenium has the X-ray K absorption edge at a wavelength of 0.979 Å and exhibits a significant anomalous signal, which can be very conveniently used for phasing by the Multi- or Single-wavelength Anomalous Diffraction (MAD^12^ or SAD^13^) approaches at any of the available synchrotron beam lines. Selenium can be also chemically introduced into nucleic acids^14^,^15^.

However, the tests have shown that it is possible to effectively introduce selenium into native crystals by soaking them in the presence of selenourea (SeU, SeC(NH_2_)_2_), Fig. 1. This simple compound, similar to well known urea (Supplementary Fig. 1), penetrates through the crystal solvent channels and binds to certain functions at the surface of biomolecules, in analogy to the heavy-atom or halide derivatization. The interactions of SeU at the macromolecular surface are different than of the hitherto utilized compounds. The two amide groups of SeU may serve as donors in hydrogen bonds formed with various acceptors, such as carbonyl or carboxyl functions of various amino acids, hydroxyl groups of Thr and Tyr residues or suitable atoms of nucleic acids. On the other hand, the Se atom in the central selenocarbonyl moiety of SeU may accept hydrogen bonds from various donors, provided by amides, hydroxyl groups and protonated amines. SeU can interact with solvent water molecules through both of these ways. Figure 2 illustrates some examples how SeU binds to proteins and a DNA oligomer.

The use of SeU as a provider of anomalous signal for the SAD phasing has been examined on crystals of several proteins and a nucleic acid. These crystals may either be soaked for a few minutes in the appropriate cryoprotecting solution complemented with SeU powder or a pinch of the powderized SeU may be added directly to the crystallization drops containing native crystals. The latter approach has the advantage of not diluting or significantly modifying the content and concentration of the original crystallization medium. The amount of SeU powder added into mother liquor or cryo-solution is about 5% in volume.

The SeU molecule is small, of the size smaller than most of the heavy-metal complexes used for classic derivatization of proteins, and in analogy to small halide ions, rapidly diffuse through the solvent channels of macromolecular crystals. It can be used in a wide pH range, at least from 4 to 9. To prevent SeU from the potential oxidation of the Se atom in solution, it may advisable to add a reducing agent, such as sodium sulfite (Na_2_SO_3_) or tris(2-carboxyethyl)phosphine (TCEP). The high concentrations of urea are routinely used for denaturation of proteins, but no adverse effects were observed after relatively short exposure of protein crystals to SeU.

Similar to halide ions, but opposite to heavy-atom complexes, SeU does not require any chemical modification or hydrolysis before binding to hydrophilic groups at the surface of macromolecules. In contrast to the Cl^−^, Br^−^ and I^−^ ions that can only serve as acceptors of hydrogen bonds, SeU is a potent donor of hydrogen bonds through its two amide groups. In addition, the lone electron pairs of the selenium atom may act as acceptors.

The examples shown in the Methods section ascertain that SeU can be successfully and conveniently used as a practical and easily applicable vehicle for phasing novel crystal structures of macromolecules by the SAD (or MAD) approach through the anomalous signal of selenium.

## Methods

The proteins selected for testing the SeU as a phasing vehicle were: HEW lysozyme^16^, thaumatin^17^, bovine trypsin^18^, cyan fluorescent protein (CFP)^19^ and histidinol phosphate phosphatase (HPP)^20^. In addition, a B-DNA Dickerson-Drew dodecamer (DDD)^21^ was used.

The information about proteins and their crystals selected for testing the SeU phasing is presented in Supplementary Table 1.

### SeU sub-packaging

SeU was purchased from Sigma (98%, Product Number: 230499) packed in brown glass bottle under argon due to air and moisture sensitivity. The shell around SeU was already oxidized to dark -selenium^22^. SeU was subpackaged into 1.5 mL Eppendorf tubes, where each tube only contained a small amount of SeU crystalline powder in order to reduce the frequency of exposing to the environment.

### Protein expression, purification, and crystallization

#### Lysozyme

Lyophilized powder of lysozyme from chicken egg white was obtained from Sigma (Product Number: L4919) and used without further purification. Lysozyme was dissolved in 10 mM pH 4.6 citrate buffer at a concentration of 40 mg/mL and mixed 1:1 with the well solution consisting of 25% (w/v) PEG3350 and 50 mM citrate buffer pH 4.0. Crystals appeared after two days at room temperature in sitting-drops. The powder of SeU was picked up by 2 μL pipette tip and directly transferred into the mother liquor. After 10 min, the color of mother liquor turned to slightly brown due to the oxidation of SeU (Supplementary Fig. 2). A crystal was fished out and washed with the well solution containing additional 20% (v/v) 2-methyl-2,4-pentanediol (MPD), then flash frozen in liquid nitrogen.

#### Thaumatin

Thaumatin was purchased from Sigma (Product Number: T7638) and used without further purification. Thaumatin was dissolved in 50 mM HEPES buffer pH 7.0 at a concentration of 35 mg/mL. Crystals appeared after two days after mixing 1 μL protein solution and 1 μL well solution containing 750 mM sodium/potassium tartrate, 100 mM citrate buffer pH 6.5 using hanging-drop vapor diffusion method at room temperature. The SeU powder was added into the cryo-solution consisting of well solution supplemented with 20% (v/v) glycerol and 50 mM Na_2_SO_3_. Native thaumatin crystals were transferred from mother liquor into cryo-solution containing SeU and Na_2_SO_3_ and soaked for 5 min, then vitrified in liquid nitrogen.

#### Trypsin

Bovine trypsin was purchased from Sigma (Product Number T9935) and used without further purification. The complex of trypsin and benzamidine contained 30 mg/mL trypsin and 5 mg/mL benzamidine in 50 mM Tris-HCl buffer pH 7.0. Crystals appeared after three days using hanging-drop vapor diffusion method at room temperature after mixing the trypsin-benzamidine complex with reservoir consisting of 20% (w/v) PEG8000, 200 mM ammonium sulfate, 100 mM citrate buffer pH 6.5 at 1:1 ratio. The SeU powder was added into cryo-solution consisting of 30% (w/v) PEG3350, 20% (v/v) MPD, 50 mM Tris-HCl pH 7.0, and 50 mM Na_2_SO_3_. Crystals were fished out from the drop and transferred into cryo-solution, soaked for 5 min, then flash frozen in liquid nitrogen.

#### CFP

The clone for CFP overexpression was acquired from the Midwest Center for Structural Genomics, with the CFP open reading frame ligated into pMCSG68 vector. The pMCSG68 vector introduces the N-terminal His6-tag, followed by the Tobacco Etch Virus (TEV) protease cleavage site preceding the genuine sequence of the expressed protein. Overexpression was carried out in BL21 Gold E. coli cells (Agilent Technologies). The bacteria were cultured with shaking at 210 rpm in LB media supplemented with 150 μg/mL ampicillin at 37 °C until the A600 reached 1.0. The cultures were cooled down to 18 °C and the CFP production was induced by addition of isopropyl-D-thiogalactopyranoside to the final concentration of 0.5 mM. The protein expression was carried out for 18 h and then the cultures were centrifuged at 3,500 g for 20 min at 4 °C. Cell pellet from 1 L culture was resuspended in 35 mL of binding buffer (50 mM Tris-HCl pH 8.0; 500 mM NaCl; 20 mM imidazole; 1 mM TCEP) and stored at −80 °C. The samples were thawed and the cells were disrupted by sonication using bursts of total duration of 4 min, with appropriate intervals for cooling. Cell debris was pelleted by centrifugation at 25,000 g for 30 min at 4 °C. The supernatant was applied to a column packed with 10 mL of HisTrap HP resin (GE Healthcare), plugged into the Vacuum Manifold (Promega) connected to a vacuum pump. After binding, the column was washed five times with 50 mL of the binding buffer and His6-tagged CFP was eluted with 20 mL of elution buffer (50 mM Tris-HCl pH 8.0; 500 mM NaCl; 300 mM imidazole; 1 mM TCEP). The His6-tag was cleaved with TEV protease (final concentration 0.2 mg/mL) and the excess of imidazole was removed by dialysis (overnight at 4 °C) at the same time. The solution was mixed with HisTrap HP resin to remove the cleaved His6-tag and the remaining His6-tagged TEV protease. The flow-through was collected, concentrated to 3.5 mL and applied on a HiLoad Superdex 200 16/60 column (GE Healthcare) equilibrated with a buffer composed of 25 mM Tris-HCl pH 8.0, 200 mM NaCl and 1 mM TCEP. Homogenous fractions of CFP monomer was collected and concentrated to 15.6 mg/mL. Crystallization screening was carried out by robot (Mosquito) using sitting-drop vapor diffusion method. Crystallization was manually optimized by sitting-drop method at room temperature. The best crystals were obtained after four days at room temperature by mixing 1uL CFP solution with 1 μL well solution consisting 16% (w/v) PEG 3350, 50 mM citric acid, and 50 mM bis-tris propane buffer pH 5.0. The SeU crystalline powder was added into cryo-solution containing 30% (w/v) PEG 3500, 20% (v/v) MPD, and 50 mM potassium phosphate buffer pH 7.5, then CFP crystals were transferred from mother liquor to cryo-solution. After a 5 min soak with SeU, crystals were flash frozen in liquid nitrogen.

#### HPP

The HPP protein expression, purification, and crystallization were described elsewhere^20^. In short, the crystals of HPP were grown in 15% (w/v) PEG 3350, 0.2 M diammonium hydrogen phosphate buffer pH 8.0 at room temperature. Crystals appeared in one week and were kept in the hanging drop for about half year. SeU powder was directly dropped into the mother liquor with HPP crystals for 10 min, then soaked crystals were transferred to paratone-N to remove the surface water and vitrified in liquid nitrogen.

#### DDD

The B-DNA Dickerson-Drew dodecamer d(CGCGAATTCGCG)_2_ was purchased from Eurofins MWG Operon (Huntsville, USA) and used without further purification. The DDD solution at 2 mM concentration was incubated at 60 °C for 10 min, then slowly cooled down to room temperature. Crystals appeared after two days at room temperature using sitting-drop vapor diffusion method by mixing DDD solution with precipitant consisting of 40 mM sodium cacodylate pH 7.0, 12 mM spermine tetrachloride, 80 mM NaCl, 10%(v/v) MPD at ratio 1:1. The well solution contained 35% (v/v) MPD. The SeU powder was added into cryo-solution consisting 40% (v/v) MPD and 50 mM Tris-HCl buffer pH 7.0 to generate SeU saturated solution. The DDD crystals were transferred from mother liquor to the SeU saturated cryo-solution and soaked for 1 min, then rapidly transferred to a goniostat under a stream of gaseous nitrogen at 100 K delivered by an Oxford Cryosystems cryocooler at the beamline.

### X-ray diffraction data collection and processing

All diffraction data were collected at a wavelength corresponding to slightly higher energy than the as absorption edge of Se, at the SER-CAT 22-ID/BM beamlines of the Advanced Photon Source (Argonne National Laboratory, USA). The diffraction data from lysozyme, thaumatin, trypsin, and CFP crystals were collected with the 180° total rotation range, 1° per image. The diffraction data of HPP were collected with 100° range with half degree per image and DDD diffraction data were acquired with 360° range and 2° per image. All data sets were processed by *HKL-*2000 and with the “auto-correction”^23^ option in scaling. The “no merge original index” option was used to generate alternative, unmerged set of data, intended only to calculate correlation coefficient of anomalous difference for two random half set (CC_ano_) by *phenix.anomalous_signal*^24^. For lysozyme and thaumatin, the data were scaled separately within 45°, 90°, 180° rotation range to analyse the strength of anomalous signal at different multiplicity. Similarly, the data sets of trypsin and CFP were scaled with 90° and 180° rotation range. The data of HPP were only scaled with 100° rotation range. Regarding DDD, the data were scaled within 90°, 180°, and 360° rotation range. The plots of CC_ano_ versus resolution showed strong anomalous signals for data sets of lysozyme, thaumatin, trypsin, CFP, and DDD, even at low multiplicity (Supplementary Fig. 3). The anomalous signal of HPP was relatively weak. The statistics of diffraction data at maximal redundancy are listed in Supplementary Table 1.

### Substructure determination, density modification, model building and structure refinement

*SHELXD*^25^ was used for anomalous substructure determination for all data with 1,000 phase trials except for HPP with the number of trials increased to 10,000. The correlation coefficients between observed and calculated normalized anomalous differences within all data (CC_all_) and 30% of reflections which were not used during the dual-space refinement (CC_weak_) with increasing multiplicity are illustrated in Supplementary Fig. 4 and Supplementary Table 2. The substructure refinement, density modification and the initial chain tracing were carried out by *SHELXE*^25^, which provided high quality density maps clearly showing the side chain groups of the autotraced poly-Ala backbones for lysozyme, thaumatin, trypsin, and CFP. Model building was carried out by *ARP/wARP*^26^, which built more than 90% of total amino acids for lysozyme, thaumatin, trypsin, and CFP (Supplementary Table 2). Chain tracing and model building of HPP did not yield interpretable density map. After *phenix.autosol* and *phenix.autobuild*^27^, 233 residues were built out of total 277 residues of HPP. Nine base-pairs were successfully built for DDD by *phenix.autobuild* with the calculated phasing and density map from *SHELXE*. The final models were refined by *REFMAC5*^28^ with occupancies refined for each SeU molecule. Water molecules were added by *COOT*^29^. The statistics of structure refinement are also listed in Supplementary Table 1. The overall structures were illustrated by *PyMoL*^30^.

## Additional Information

**Accession codes:** Protein Data Bank (PDB) codes: 5T3F (SeU-lysozyme), 5T3G (SerU-thaumatin), 5T3H (SeU-trypsin), 5T3I (SeU-CFP), 5T3J (SeU-HPP), 5T3L (SeU-DDD).

**How to cite this article**: Luo, Z. Selenourea: a convenient phasing vehicle for macromolecular X-ray crystal structures. *Sci. Rep.* **6**, 37123; doi: 10.1038/srep37123 (2016).

**Publisher’s note**: Springer Nature remains neutral with regard to jurisdictional claims in published maps and institutional affiliations.

## Supplementary Material

Supplementary Information

## Figures and Tables

**Figure 1:**
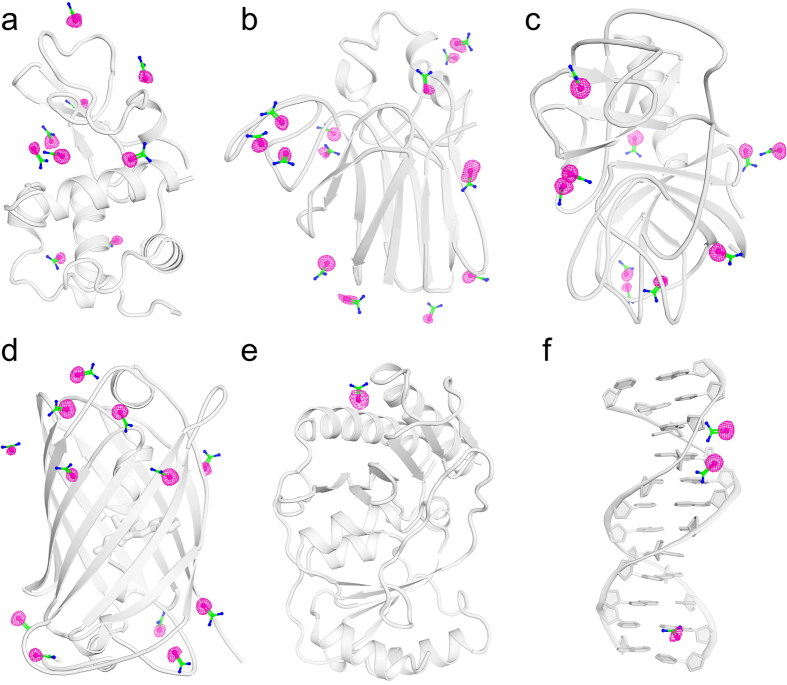
Selenourea binding sites illustrated by anomalous difference maps. (**a**) Lysozyme, 9 binding sites; (**b**) thaumatin, 13 binding sites; (**c**) trypsin, 10 binding sites; (**d**) CFP, 12 binding sites; (**e**) HPP, 1 binding site; (**f**) DDD, 3 binding sites. Macromolecules are shown as cartoon. SeU are shown as ball-and-stick with selenium colored pink, carbon colored green, and nitrogen colored blue. Anomalous difference maps are shown as pink mesh at 4 σ level.

**Figure 2:**
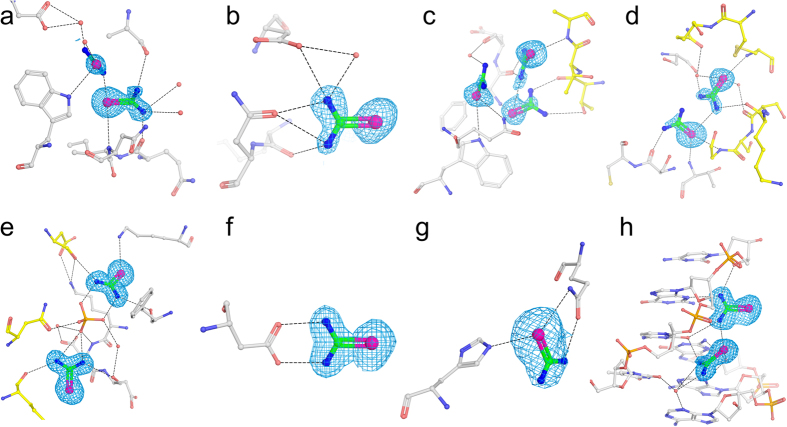
Selenourea-macromolecules interactions. (**a**) Lysozyme, sites 1 and 3; (**b**) lysozyme, site 2; (**c**) thaumatin, sites 6, 7, and 8; (**d**) trypsin, sites 4 and 5; (**e**) CFP, sites 1 and 3; (**f**) CFP, site 10; (**g**) HPP, site 1; (**h**) DDD, sites 1 and 2. Atoms and bonds are shown as ball-and-stick and valences of bonds are also illustrated. Water molecules involved in interactions are shown as red balls. Color scheme of macromolecules: C, gray or yellow (if symmetry equivalent); N, blue; O, red. 2F_o_-F_c_ density maps of SeU are shown as blue mesh at 1σ level. Hydrogen bonds are shown as black dashed lines.
