# Supplementary material for: Selenourea: a convenient phasing vehicle for macromolecular X-ray crystal structures
Source: Sci Rep. 2016 Nov 14;6:37123. doi: 10.1038/srep37123 (PMC5107899; doi:10.1038/srep37123)

Selenourea: a convenient phasing vehicle for macromolecular X-ray crystal structures

Zhipu Luo

Synchrotron Radiation Research Section, National Cancer Institute,
Argonne National Laboratory, Argonne, 60439, USA

Correspondence e-mail: luozhipu@anl.gov

Supplementary Material

Supplementary Table 1 Statistics of diffraction data and model refinement

Supplementary Table 2 Phasing and model building results

Supplementary Fig. 1 Chemical structures of urea and selenourea

Supplementary Fig. 2 Soaking crystals with selenourea powder

Supplementary Fig. 3 The correlation coefficient of anomalous difference

Supplementary Fig. 4. Anomalous substructure determination with *SHELXD*

Supplementary Table 1

Statistics of diffraction data and model refinement.

Protein		Lysozyme	Thaumatin	Trypsin	CFP	HPP	DDD
PDB code		5T3F	5T3G	5T3H	5T3I	55T3J	5T3L
MW (kDa)		14.3	22.2	23.3	26.9	30.1/4	7.2
Residues in AU		129	207	223	239	274/7	24
Solvent content (%)		37	56	46	40	61	47
Beam line		APS 22-BM	APS 22-BM	APS 22-BM	APS 22-ID	APS 22-BM	APS 22-ID
Wavelength (Å)		0.979	0.978	0.978	0.978	0.978	0.979
Crystal Sizes (mm)		0.50×0.40×0.30	0.20×0.10×0.10	0.30×0.10×0.10	0.60×0.05×0.05	0.35×0.20×0.20	0.80×0.06×0.04
Space group		$P4_32_12$	$P4_12_12$	$P2_12_12_1$	$P2_12_12_1$	$P6_222$	$P2_12_12_1$
Resolution (Å)		1.45(1.50-1.45)	1.55(1.61-1.55)	1.55(1.61-1.55)	1.60(1.66-1.60)	2.55(2.64-2.55)	1.58(1.64-1.58)
Cell	a (Å)	77.4	57.6	53.8	52.1	119.6	24.4
	b (Å)	77.4	57.6	56.6	61.5	119.6	41.5
	c (Å)	37.3	150.8	65.4	69.6	92.9	66.0
Multiplicity		13.4(13.4)	18.5(9.1)	7.3(7.3)	7.3(7.3)	11.8(12.1)	13.2(10.6)
Completeness (%)		99.9(99.9)	97.7(95.9)	100.0(100.0)	100.0(100.0)	99.9(100.0)	100.0(100.0)
$I/\sigma(I)$		42.9(2.1)	41.0(1.9)	32.3(1.7)	23.2(1.8)	24.7(1.9)	21.9(2.1)
R_{merge} (%)		5.4(130.3)	7.9(88.6)	5.7(132.4)	5.0(128.1)	10.1(122.1)	13.8(130.7)
$CC_{1/2}$		94.8(75.7)	94.5(70.3)	91.8(62.0)	91.1(59.9)	93.9(70.7)	96.4(90.3)
R factor (%)		13.4(19.0)	13.0(19.0)	13.2(20.6)	13.4(19.8)	20.4(30.9)	14.8(15.0)
R free (%)		18.4(29.1)	16.6(19.8)	18.3(25.1)	17.7(24.1)	23.7(39.1)	18.6(23.5)
Rmsd bonds (Å)		0.011	0.010	0.010	0.011	0.012	0.010
Rmsd angles (°)		1.39	1.27	1.35	1.67	1.61	1.66

Supplementary Table 2

Phasing and model building results.

Protein	Lysozyme			Thaumatin			Trypsin		CFP		HPP	DDD		
Rotation range (°)	45	90	180	45	90	180	90	180	90	180	100	90	180	360
SHELXD														
CC_{all} (%)	37.5	42.6	46.0	33.3	38.1	44.4	34.2	33.5	40.1	34.5	31.5	32.9	36.6	38.7
CC_{weak} (%)	22.4	24.6	27.5	19.6	21.3	24.9	11.4	15.0	22.0	19.7	13.8	20.1	19.9	22.1
SHELXE														
Sites	10	11	11	11	12	13	10	17	14	13	1	2	3	3
mapCC (%)	71.9	78.3	80.0	86.6	90.3	89.4	73.7	78.3	72.9	80.7	75.9	58.2	76.3	88.5
CC (E_{obs} vs E_{cal})(%)	30.6	38.6	37.3	41.0	41.3	40.8	32.0	35.8	37.9	41.2	11.4			
Chain_tracing	97	124	123	193	195	191	176	186	201	218	88			
ARP/wARP														
Model building	94	126	126	200	200	200	213	215	220	221				
Phenix.autosol/autobuild														
No. of Residues											233	17	8	21
R_{work} (%)											24.7	45.4	47.8	33.3
R_{free} (%)											30.2	53.1	56.6	35.3

Supplementary Fig. 1 Chemical structures of urea (left) and selenourea (right).

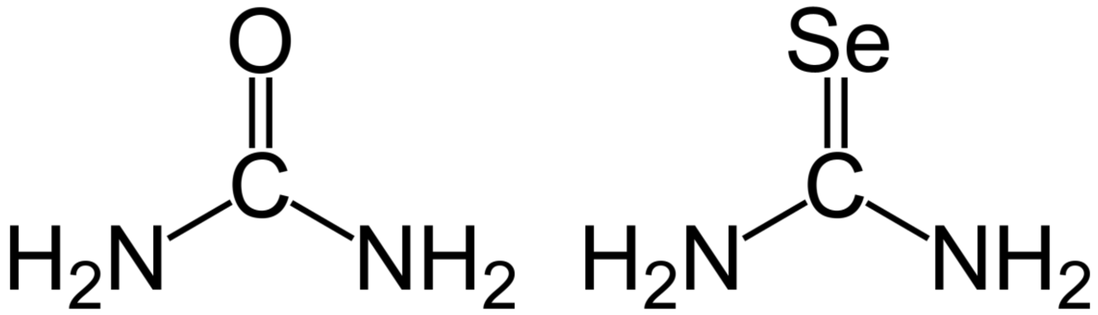

Supplementary Fig. 2 Soaking crystals with selenourea powder. Soaking lysozyme (a), thaumatin (b), and trypsin (c) crystals with SeU by directly adding the SeU crystalline powder into the crystallization drops containing native crystals for 10, 5, and 5 min respectively. The crystalline powder of SeU was marked by red oval.

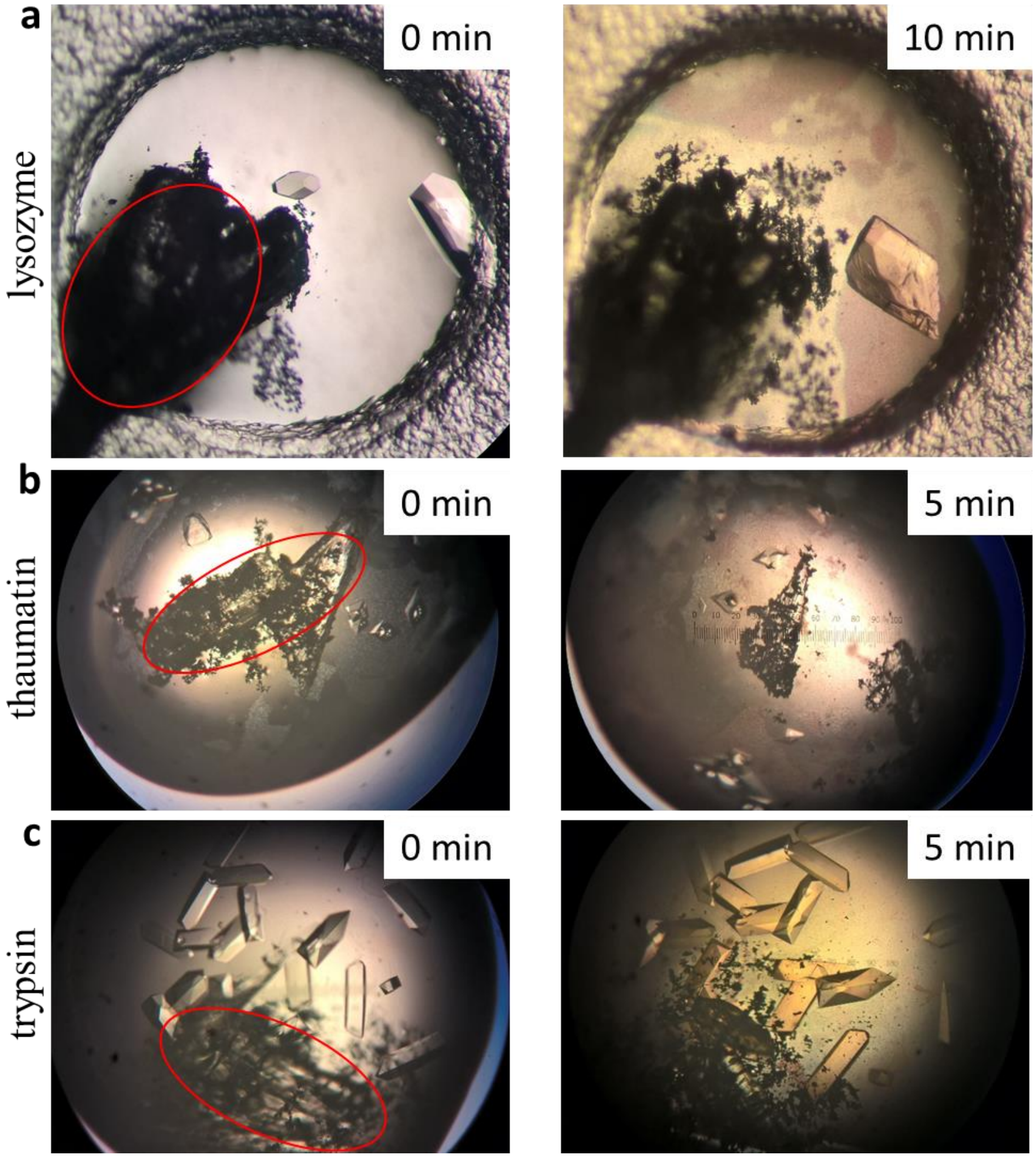

Supplementary Fig. 3. The correlation coefficient of anomalous difference for two random half set (CC_{ano}) versus resolution with different total crystal rotation ranges of lysozyme (a), thaumatin (b), trypsin (c), CFP (d), HPP (e), and DDD (f) diffraction data. CC_{ano} for each crystal data at 30% were shown as dash lines.

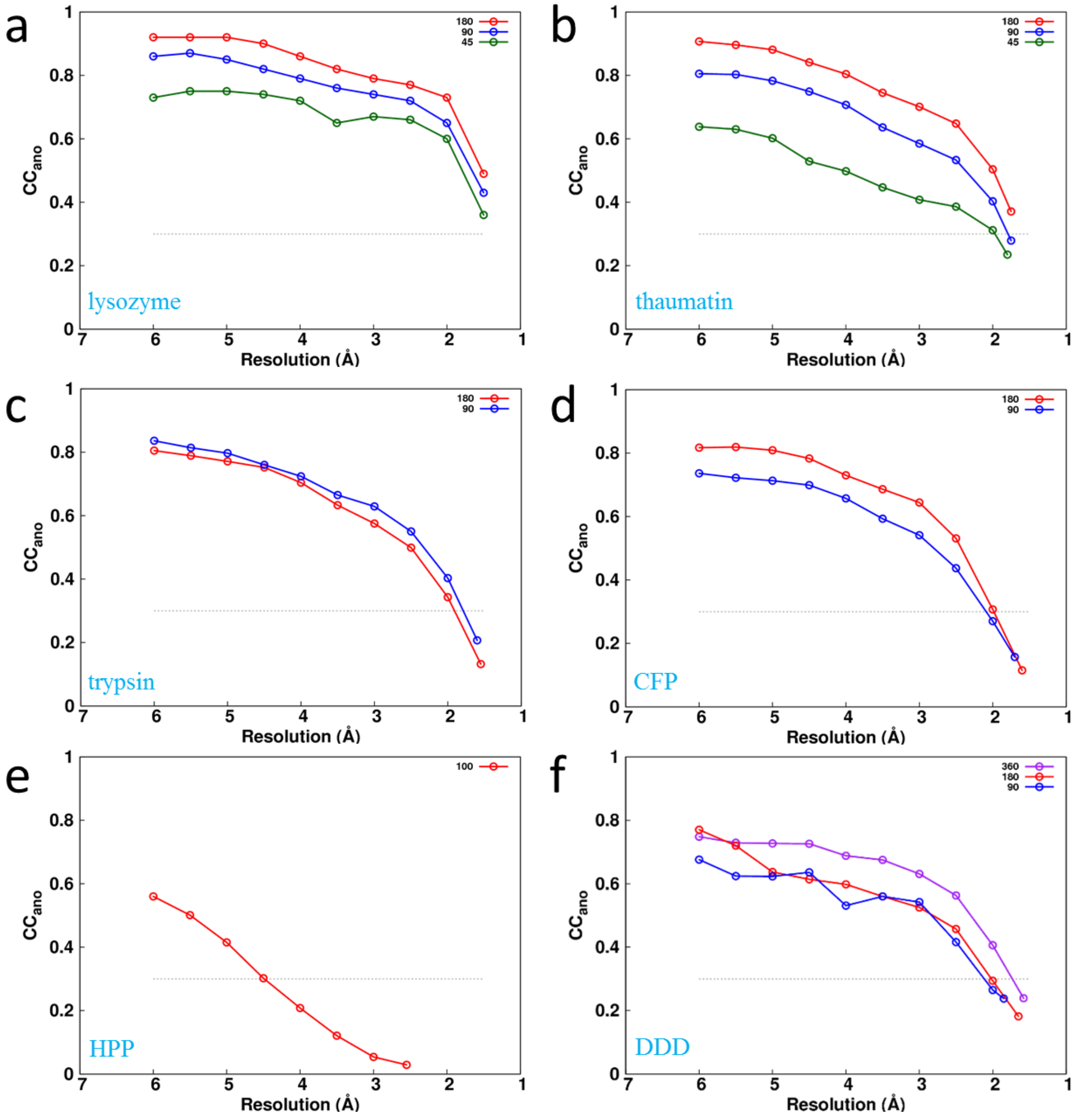

Supplementary Fig. 4 Anomalous substructure determination with *SHELXD* characterized by CC_{all}/CC_{weak} plots with 1000 trials for lysozyme(a), thaumatin (b), trypsin (c), CFP (d), DDD(f) and 10000 trials for HPP(e). The crystal rotation range and resolution cut-off employed in *SHELXD* were labeled on each plot.

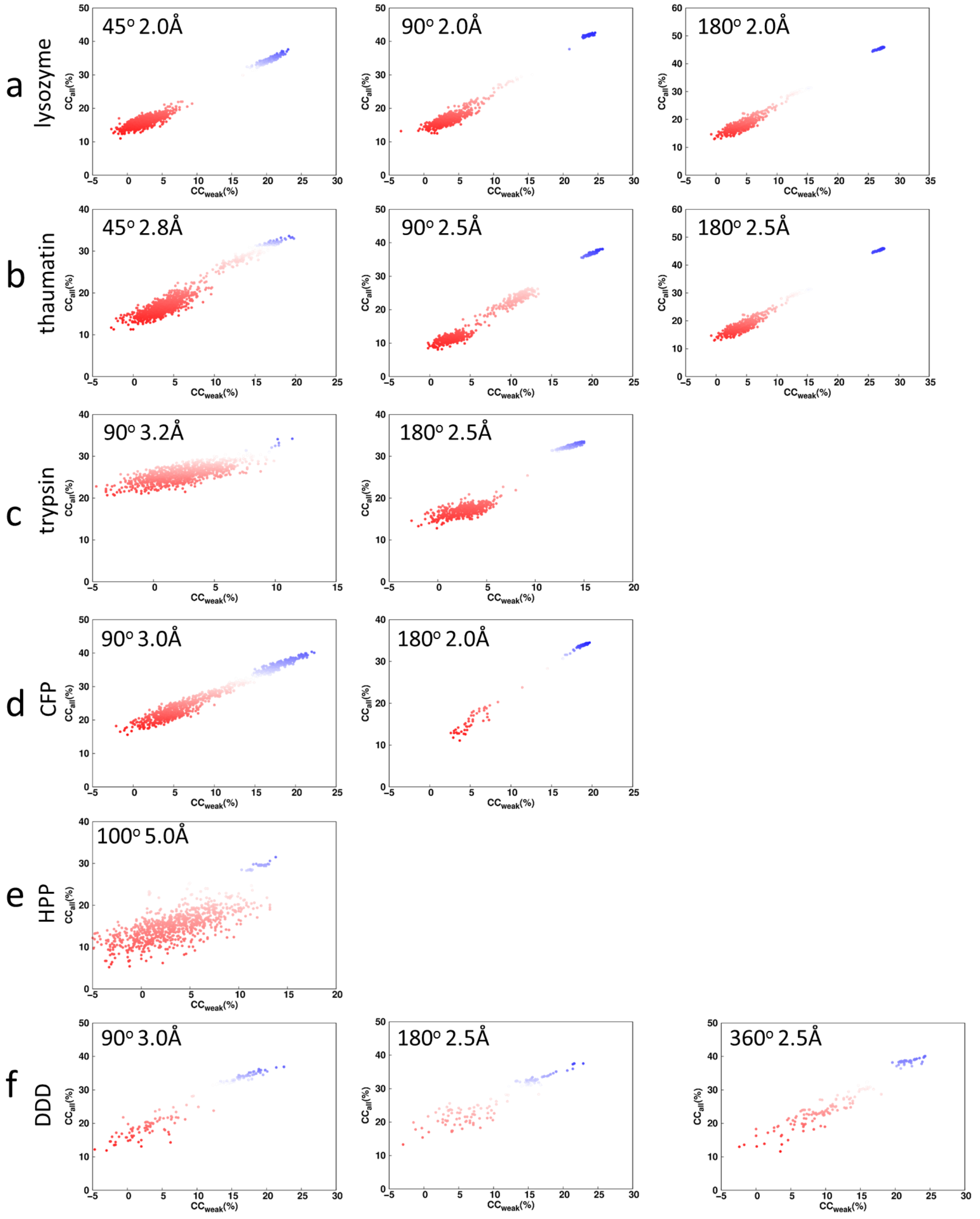

Supplement: Supplementary Information [file srep37123-s1.pdf]
